# The HMOX1 Pathway as a Promising Target for the Treatment and Prevention of SARS-CoV-2 of 2019 (COVID-19)

**DOI:** 10.3390/ijms21176412

**Published:** 2020-09-03

**Authors:** Neelu Batra, Cristabelle De Souza, Jyoti Batra, Alan G. Raetz, Ai-Ming Yu

**Affiliations:** 1 Department of Biochemistry and Molecular Medicine, UC Davis School of Medicine, Sacramento, CA 95817, USA; nbatra@ucdavis.edu (N.B.); cmdesouza@ucdavis.edu (C.D.S.); agraetz@ucdavis.edu (A.G.R.); 2 Department of Internal Medicine, University of New Mexico Cancer Center, University of New Mexico School of Medicine, Albuquerque, NM 87131, USA; 3 Gladstone Institute, San Francisco, CA 94158, USA; jyoti.batra@gladstone.ucsf.edu

**Keywords:** SARS-CoV-2, HMOX1, ORF3a, HMOX1-ORF3a, natural compounds, anti-viral therapy

## Abstract

The coronavirus disease of 2019 (COVID-19) or severe acute respiratory syndrome coronavirus 2 (SARS-CoV-2) infection is a global pandemic with increasing incidence and mortality rates. Recent evidence based on the cytokine profiles of severe COVID-19 cases suggests an overstimulation of macrophages and monocytes associated with reduced T-cell abundance (lymphopenia) in patients infected with SARS-CoV-2. The SARS-CoV-2 open reading frame 3 a (ORF3a) protein was found to bind to the human HMOX1 protein at a high confidence through high-throughput screening experiments. The HMOX1 pathway can inhibit platelet aggregation, and can have anti-thrombotic and anti-inflammatory properties, amongst others, all of which are critical medical conditions observed in COVID-19 patients. Here, we review the potential of modulating the HMOX1-ORF3a nexus to regulate the innate immune response for therapeutic benefits in COVID-19 patients. We also review other potential treatment strategies and suggest novel synthetic and natural compounds that may have the potential for future development in clinic.

## 1. Introduction

The ongoing coronavirus disease of 2019 (COVID-19) or severe acute respiratory syndrome coronavirus 2 (SARS-CoV-2) infection poses an unprecedented threat to public health as a global pandemic accompanied with high incidence of mortalities [1]. Most of the COVID-19 fatalities are due to respiratory failure caused by acute respiratory distress syndrome (ARDS) [2]. Characterized by clinical symptoms such as fevers or chills, shortness of breath, muscle, and body aches [3], this flu-like disease has resulted in staggering outcomes with multiple mechanisms of action. In addition, recent studies have shown that fatalities of patients on mechanical ventilation over the age of 65 was 97.2%, significantly higher compared to 76.4% for ages ranging from 18 to 65 [4]. Owing to a large spike recently, especially among the elderly population, it has become imperative to identify new safe and effective therapeutic strategies that include, but are not limited to, anti-viral therapy, vaccines and immune-modulating drugs [5].

Current treatment research strategies for COVID-19 are investigating several novel frontiers of therapeutics, such as the anti-viral drugs *remdesivir*, *favilavir*, the anti-malarial *hydroxychloroquine*, the anti-HIV drugs *lopinavir*, *ritonavir* and ACE2 inhibitor *APN01* [6,7,8]. It is also promising that that there is an ongoing phase 3 clinical trial with the investigational vaccine mRNA-1273 in the United States [9]. However, in spite of the immense investigational efforts being made for treating patients with COVID-19, additional therapeutic strategies are needed. Passing regulations required by the US Food and Drug Administration (FDA) and proving safety with efficacy in multi-arm clinical trials will take longer periods of time, thus delaying treatment to patients. Owing to restricted treatment modalities, it is imperative to focus on repurposed drugs and natural compounds that target protein interactors of the SARS-CoV-2 protein. It would be interesting to note potential molecular therapeutics that could modulate the HMOX1 pathway to enhance therapeutic intervention and control the cytokine cascade commonly observed in SARS-CoV-2 patients worldwide. These treatment options could be especially beneficial to developing countries in the subcontinents of Asia and Africa, where mass vaccination and medical facilities are limited. In this article, we enlist the existing treatment options that are being investigated as frontline therapy for the treatment of SARS-CoV-2. We also throw light on the benefits of naturally occurring compounds and potential mechanisms that can be elucidated to understand the molecular targets of the HMOX1 pathway and other pathways that are altered in COVID-19 patients.

## 2. Life Cycle and Infectious Process of the SARS-CoV-2 Virus

**Figure 1:**
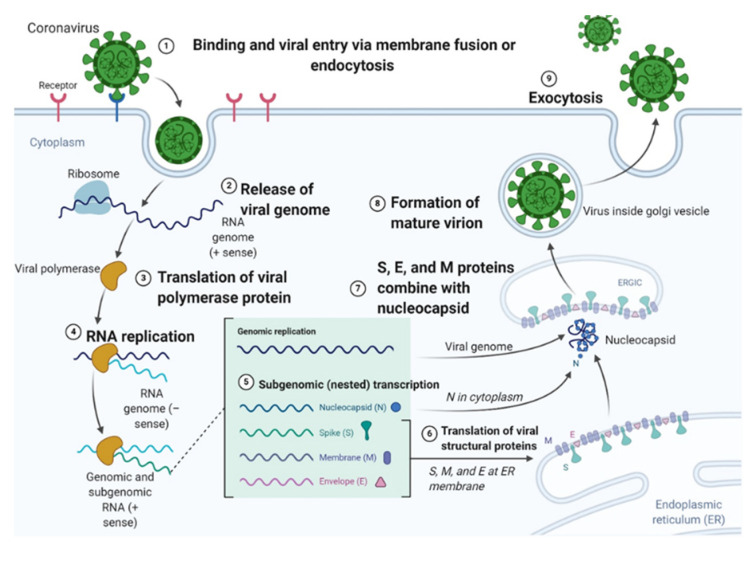
Schematic illustration of the coronavirus entry and replication cycle within the human host. (Image modified from BioRender Templates, also found in [13]).

The SARS-CoV-2 virus is part of a family of enveloped positive-sense RNA viruses. Morphologically distinct with spikes on the viral surface, it has a distinct molecular replication strategy (Figure 1). The entry of the virus into the host cell can occur either via endocytosis or plasma membrane fusion. Irrespective of the mode of entry, two spike proteins of the virus called S1 and S2 attach to the membrane of the host cell and use the angiotensin-converting enzyme 2 (ACE2) receptor for entry. If entry is via the endosomes, cathepsin L activates the spike proteins which can also be activated by the cellular serine protease TMPRSS2. However, the membrane fusion entry path would be most efficient for viral entry owing to its decreased potential to trigger a host cell immune cascade. After entry, the viral RNA is released as shown. This viral RNA is then translated and replicated. The RNA-dependent RNA polymerase (RdRp) would bring about replication of the RNA belonging to the viral structural protein. The viral structural proteins S1, S2, envelope protein (E) and membrane protein (M) are translated by the rough endoplasmic reticulum (RER). The translated proteins are then released on the surface of the RER in preparation for virion assembly and they undergo a nested sub genomic transcription. The nucleocapsid proteins (N) remain in the cytoplasm since their assembly is from genomic RNA. The N proteins fuse with the virion precursor which is transported from the endoplasmic reticulum (ER) through the Golgi apparatus to the cell surface via small vesicles. The assembled virions are then released extracellularly through exocytosis [10,11,12].

## 3. Current Treatments for SARS-CoV-2 Patients

Currently, while there is one investigative vaccine in Phase 3, there do not exist any clinically approved anti-viral therapeutics or vaccines. Most treatment modalities focus on overcoming the challenges of respiratory failure and involve mechanical ventilation. Although there are some commonly known mechanisms of virus infection and replication in the host system (Figure 1), SARS-CoV-2 infection has multiple undiscovered pathways responsible for molecular pathogenesis. Elucidating the mechanisms underlying virus infection and immune disruption is of utmost importance. The knowledge gap that currently exists pertaining to the molecular underpinnings of SARS-CoV-2 infection provides a challenge to the field of drug discovery and molecular therapeutics. Even though drugs such as *hydroxychloroquine* and *remdesivir* have shown promise, several multi-arm clinical trials would be necessary to initiate clinical use. SARS-CoV-2 mainly uses ACE2 as its receptor, causing several research groups to study strategies to alter the binding affinity of the virus to the enzyme [14]. COVID-19 pathogenesis has been attributed to an excessive inflammatory response, cytokine storm and immune modulated tissue damage [15,16]. Of note, interleukin 6 is seen to be upregulated in COVID-19 patients with severe disease and several groups are currently looking at the potential benefits of using IL-6 inhibitors to ameliorate extreme damage to lung tissue. While IL-6 drugs such as *tocilizumab* do show some clinical efficacy, other studies reported that IL-6 inhibitors could be associated with secondary infections or even toxic responses in patients [17]. Irrespective of the current consensus, it is known that respiratory distress, sepsis, thrombosis and lung damage observed in some critically ill patients are all signs of an overactive cascade of dysregulated immune mediating molecules like cytokines [18]. Some success stories related to IL-6 inhibition as a treatment measure suggest possible therapeutic approached targeting IL-6 intervention. Dr. Ryan Padgett was one of the first frontline health-care workers in Washington to test positive for SARS-CoV-2. He successfully responded to infusions of the rheumatoid arthritis drug *Actemra*, which is an inhibitor of the IL-6 receptor, one of the several key regulators of the SARS-CoV-2 cytokine storm [19,20,21]. However, *Actemra* must be prescribed with caution as it could block the patient’s innate immune response. Details of the drug have been provided (Table 1).

Another investigational drug worth mentioning is the broad spectrum anti-viral drug *remdesivir*. A previous study has demonstrated the ability of *remdesivir* to inhibit SARS-CoV-2 [22]. *Remdesivir* is a nucleoside analogue pro-drug with unique structural features that allow high concentrations of the active triphosphate metabolite to be delivered intracellularly. It evades proofreading to successfully inhibit viral RNA synthesis and has demonstrated potent antiviral activity against β-coronaviruses, including SARS-CoV-2 in both in vitro and in animal models [8,23,24,25,26,27,28,29,30]. These data, coupled with early safety data from clinical experience in Ebola virus infection 5, provide strong rationale for prioritizing testing of *remdesivir* in COVID-19 clinical trials. With at least six *remdesivir* randomized-controlled trials currently underway worldwide, there is reason to be optimistic of its therapeutic efficacy. Currently, several ongoing phase 3 clinical trials for *remdesivir* are underway [31]. However, *remdesivir* has proven effective in some patients, but not all [32]. Consequently, although the FDA has not yet approved it, emergency use authorization for *remdesivir* has been granted (Table 1). Scientists are also considering repurposing drugs used for other diseases such as *tamiflu*, *hydroxycholoquine*, *azithromycin*, ands *lopinavir/ritonavir* for their effect in treating SARS-CoV-2 (Table 1).

## 4. COVID-19 Related Thrombosis, Sepsis and Fibrinogenesis

A previous report showed that SARS-CoV-2 patients display abnormal thrombosis (blood clotting) in addition to significantly elevated abnormal D-dimer levels [31]. It is hypothesized that the virus could directly target blood vessels or could be an indirect result of a cytokine storm [64]. This could also explain the acute kidney injury seen in several patients because of the cytokine storm response to infection. It was found that several patients with severe SARSCoV-2 had coagulation abnormalities that are associated with an increased risk of death [65]. A recent report suggested that a considerable fraction of patients with severe SARS-CoV-2 infection develop venous and arterial thromboembolic complications [66]. Moreover, one study demonstrated that all COVID-19 patients have elevated fibrinogen levels on hospital admission [65]. Another interesting study found that increased IL-6 levels found in all COVID 19 patients correlate with increased fibrinogen levels, confirming the link between inflammation and procoagulant changes [66]. Hospitals in France reported multiple cases of deep vein thrombosis associated with significantly increased inflammatory response among SARS-CoV-2 patients. High dimerized plasmin fragment D (D-dimer) levels, procoagulant changes in coagulation pathways and an elevated rate of venous and arterial thrombotic events were reported among patients with severe SARS-CoV-2 [67]. All the above findings lead to the fact that COVID-19 infection results in an uncontrolled immune response. Identifying pathways to regulate this response is of immediate importance.

## 5. Role of HMOX1 in Thrombosis, Fibrinolysis and Sepsis

Heme oxygenase (HMOX1) is a key enzyme that catalyzes the rate-limiting first step in the heme degradation process, generating carbon monoxide, ferrous and biliverdin, and therefore HMOX1 has a cytoprotective role as excess free heme has been shown to induce apoptosis [27]. HMOX1 is expressed at high levels in the lungs and has been shown to mediate anti-inflammatory effect of interleukin-10 (IL-10) in mice [68,69]. Given these functions of HMOX1, it has been implicated in a variety of pathological states, including myocardial infarction, diabetes, chronic obstructive pulmonary disease (COPD) [69,70]. Upregulation of HMOX1 has been shown to have a protective role against the oxidative stress produced upon HIV, DENV, HCV, and IAV infections [71]. As aggressive inflammatory responses in the respiratory tract are strongly implicated in disease severity upon SARS-CoV-2 infection, further exploration of HMOX1 function during virus infection will help to elucidate mechanisms of viral pathogenesis and potential treatments.

High-throughput genomic analyses of patients suffering from SARS-CoV-2 has provided growing evidence to suggest a sub-group of the affected population suffering from an inflammation-mediated cytokine storm. The cytokine storm, or cytokine release syndrome (CRS), is a systemic inflammatory response characterized by the innate immune system releasing a cascade of cytokines such as interferons, interleukins and chemokines, among others, thus overwhelming the host immune response leading to death [5,23,24,72]. This massive cytokine boost promotes high levels of interleukin-6 (IL-6) production, increased levels of clotting factors and fibrinogen leading to significantly higher rates of thrombosis in COVID-19 patients [25].

Since the SARS-CoV-2 S protein binds to the human angiotensin-converting enzyme (ACE-2), many studies are currently focusing on identifying inhibitors of this interaction as potential therapeutics [8]. However, recent studies based on protein–protein interactions have demonstrated alternative pathways that could prove promising targets for therapeutic intervention or drug repurposing [26]. The SARS-CoV-2 open reading frame 3 a (ORF3a) protein was found to bind to human HMOX-1 protein with high confidence using affinity tag purification coupled to mass spectrometry (AP-MS) in an unbiased search in human cells [26]. Given the central role of inflammation in severe COVID-19 cases, this binding interaction is promising because HMOX-1 activity reduces inflammation and tissue damage [27] via the NLRP3 pathway [73,74]. This is of interest because the ORF3a in SARS-CoV-2 has been shown to directly activate the NLRP3 inflammasome pathway [28,75].

Furthermore, a number of studies that have examined the cytokine profiles of severe COVID-19 cases suggested an overstimulation of macrophages and monocytes is associated with reduced T-cell abundance (lymphopenia) [29], although the exact mechanism that causes this dysregulation has yet to be identified. Importantly, macrophage differentiation into sub-classes termed M1 and M2 is mediated by the activity of HMOX-1 [30]. Surprisingly, M2 macrophages, which are considered an anti-inflammatory phenotype that inhibit T-cell activation, are upregulated in severe COVID-19 cases despite the high levels of inflammation present [29], and M2 macrophage dysfunction is associated with severe COVID-19 inflammation [26].

One simple explanation is that the anti-inflammatory activity of HMOX1 in M2 macrophages is directly inhibited by ORF3a binding. Alternatively, ORF3a binding to HMOX1 may allow viral particles to preferentially target M2 macrophages, and this could lead to several different outcomes. The ORF3a is an ion channel [76] that has a key role in viral particle release [77] and mediates both apoptotic [78] and necrotic [79] cell death. Thus, HMOX1 binding could inhibit the cytotoxic activity of the ORF3a protein in M2 macrophages, whereas in the pro-inflammatory M1 macrophages, with low levels of HMOX1, unbound ORF3a is activated and induces cell death, favoring the survival of M2 macrophages, which suppresses T-cell activation and thus allows the virus to evade T cell-mediated cell death. M2 macrophages could then act as a reservoir for viral particle production, driving further tissue destruction.

Interestingly, previous studies demonstrated that stimulation of HMOX1 production inhibits the platelet-dependent thrombus formation [80]. Likewise, increased HMOX1 expression in response to oxidative stress may represent an adaptive response mechanism to down-regulate platelet activation under prothrombotic conditions [80]. Another study showed direct evidence for a protective role of HMOX1 against thrombosis and reactive oxygen agents during vascular damage and inflammation. The induction of HMOX1 was shown to be of potential benefit in the prevention of thrombosis associated with inflammation and vascular oxidant stress [81]. Additionally, other groups have demonstrated that the products of HMOX1 possess antithrombotic properties, and impairment of HMOX1 activity might contribute to thrombus formation [82]. HMOX1 is the rate-limiting enzyme of heme degradation, which leads to the cleavage of the heme ring at the alpha methene bridge to form carbon monoxide, ionic iron, and biliverdin, which is further converted to bilirubin immediately by another enzyme, biliverdin reductase. We can see that with the pro-oxidant heme conversion to antioxidant bilirubin, HMOX-1 acts as a major antioxidant [83]. Studies showed that systemic induction of HMOX1 and bilirubin delays in vivo microvascular thrombus formation, most likely caused by a reduction in endothelial P-selectin [84]. Carbon monoxide is also shown to exert anticoagulant effects by influencing platelet aggregation [85], fibrinolysis [86] and has a role in maintaining the integrity of the vessel wall [87,88]. The fact that SARS-CoV-2 causes thrombosis, which in turn leads to patient death and the antithrombotic effects of HMOX1, clearly supports the idea that induction and upregulation/induction of HMOX1 might help to decrease thrombosis and reduce the severity of SARS-CoV-2 infection.

Furthermore, studies reported that sepsis observed in some critically ill SARS-CoV-2 patients could be resulting from an overactive immune system. Sepsis is a leading cause of death worldwide and leads to a hyper-inflammatory response, resulting in multi-organ failure. Briefly sepsis leads to red blood cell lysis that releases hemoglobin. Oxidation of hemoglobin releases free heme into the circulatory system. Proinflammatory mediators are induced by free heme, which can lead to tissue injury or cell death. HMOX1 is the enzyme responsible for heme scavenging in sepsis. Importantly, during sepsis the cytoprotective enzyme HMOX1 is upregulated and thus helps in combating sepsis-induced tissue injury. Additionally, HMOX1 plays an important role in protection from polymicrobial sepsis [89]. The HMOX1 byproduct, carbon monoxide increases phagocytic activity, thereby enhancing bacterial clearance [89,90]. Moreover, the HMOX1 product biliverdin leads to an increased expression of the anti-inflammatory mediator IL-10 and reduces expression of proinflammatory mediators such as IL-6 and MCP-1 [91]. An in vivo study report demonstrated that hemin, which is an inducer of HMOX1, decreases IL1b and IL-18 secretion, and protects from sepsis-induced acute lung injury by inhibiting the excessive inflammatory response [92]. An additional study demonstrated in vivo that HMOX1/CO plays a critical role in inhibiting LPS-mediated sepsis and pro-inflammatory cytokine production [93]. Hepatic injury caused by sepsis was also protected in mouse models via HMOX1-induced autophagy [94]. In a nutshell we can say that HMOX1 plays a protective role against polymicrobial sepsis, acute inflammation response and thrombosis that are seen in critical SARS-CoV-2 patients. Therefore, agents upregulating the HMOX1 pathway could prove to be potential therapeutic or preventive agents for SARS-CoV-2.

## 6. Small Molecules for Host Directed Therapy of SARS-CoV-2

The SARS-CoV-2 viral genome has 13 open reading frames (ORFs) obtained from nine sub-genomic RNAs. The viral proteins encoded are of four structural types: spike (S), envelope (E), membrane (M) and nucleocapsid (N). The genetic composition of the SARS-CoV-2 virus shares some similarities with the SARS-CoV-2 virus. The ORF1a/1b encodes polyproteins that are ultimately processed to form 16 non-structural proteins (Nsp’s) [26,89]. In addition to HMOX1, the SARS-CoV-2 viral protein showed high interaction with other human proteins that were essential to biological processes such as DNA replication, epigenetic regulation, vesicle trafficking, RNA processing and regulation, ubiquitin ligases and nuclear transport. Importantly, since the SARS-CoV-2 viral envelope interacts with bromodomain proteins, such as BRD2 and BRD4, several bromodomain inhibitors such as *GSK1210151A*, *GSK525762*, *OTX-015*, *TEN-010*, *CPI-203*, *CPI-0610* show great potential in inhibiting the replication potential of the virus [95,96,97,98,99,100,101,102,103]. Previous studies have looked at the potency of drugs such as *zotatifin*, an inhibitor of a translation initiation factor, PB28, an agonist of the sigma-2 receptor and hydroxychloroquine, a quinoline derivative used as an anti-malarial, *zotatifin* was found to have the highest potency with an IC_50_ of 37 nanomolar [26]. Another exciting avenue to pursue is the use of inhibitors of messenger RNA translation. In 2012, the FDA approved the first inhibitor of RNA translation called *omacetaxine mepesuccinate* which is a tyrosine kinase inhibitor (TKI) for the treatment of chronic myelogenous leukemia (CML) [103]. Additionally, other serine/threonine kinase inhibitors like the mammalian target of rapamycin (mTOR) specific FDA approved drugs *temsirolimus* and *everolimus* could also be potential options for testing [104,105]. It is important to note that previous studies specifically identified sigma 1 and sigma 2 receptors to be important binding targets of the SARS-CoV-2 viral protein. The sigma-1 and sigma-2 receptors are resident proteins in the endoplasmic reticulum (ER) and have historically been associated with neurological disorders, HIV, and cancer. It is also widely known for its interaction with ion channels on the plasma membrane of cells [106]. They are found to modulate potassium and ion channels and play dual roles of molecular chaperones and receptors [104,106]. Sigma receptor antagonists like *rimcazole*, *BD-1047*, *BD1063* and *siramesine* have previously demonstrated potent activities for the treatment of cancer and HIV [105]. Thus, novel modulators as well as conventional antagonists of the sigma receptor proteins could serve as novel frontiers for drug discovery or repurposing in the setting of COVID-19.

## 7. Natural Antiviral Agents that Upregulate the HMOX1 Pathway

In addition to synthetic molecules that are potential treatment strategies for SARS-CoV-2 viral infection, it is important to note some compounds/extracts obtained from natural plants that could show promise for therapy. One such natural plant is the neem plant (*Azardirachta indica*), known for its anti-inflammatory, antioxidant, antimalarial, antiarthritic, antipyretic, hypoglycemic, antigastric ulcer, antifungal, antibacterial, and antitumour activities or immune-stimulating properties [107,108,109,110,111,112]. There are currently more than 140 biologically active compounds including *Nimbolide* that has been shown to be a unique druggable modality especially in cancer pathogenicity [113]. Nimbolide is a limonoid tetranortriterpenoid with an α,β-unsaturated ketone system and a δ-lactone ring isolated from *Azadirachta indica*. A study showed that aqueous neem bark extract (NBE) preparation from remarkably blocked herpes simplex virus 1 (HSV-1) entry into natural target cells at concentrations ranging from 50–100 µg/mL. [114]. Many reports determined that neem extracts significantly inhibited various viruses such as coxackie B group virus, poliovirus, dengue virus and HIV at early steps of viral genome replication. In vitro and in vivo studies have described the inhibitory potential of crude aqueous extract of neem leaves and pure neem compound *(Azadirachtin)* on the replication of dengue virus type-2 [115]. Azadirachtin is a triterpenoid found in need tree seeds. Studies have reported the antiviral properties of *Azadirachta indica* polysaccharides for poliovirus in vitro [116], by mechanistically inhibiting initial stages of viral replication. The neem extract was also found to act as a viricidal agent against the coxsackie virus B-4 [117]. Water-extracted polysaccharides from neem leaves exerted anti-bovine herpesvirus type 1 (BoHV-1) activity and a fractionated acetone-water neem extract, IRAB is being marketed as a drug against HIV, malaria, and cancer in Nigeria under the trade name IRACAP [118]. Additional groups have shown the antiviral activity of neem seed kernel extracts in vitro against duck plague virus (Table 2) [119].

## 8. Upregulation of Protein and mRNA Levels of HMOX1 Using Natural Compounds and Clinically Available Therapeutics

While the neem plant may not be the only natural compound that has therapeutic potential for its anti-viral properties, previous reports showed that the neem leaf extract upregulates the HMOX1 protein and mRNA expression [121]. One such study reported a significant increase in the HMOX1 protein level in C4-2B and PC-3M-luc2 prostate cancer cells after 24 and 48 h of treatment with the EENL (ethanolic extract of neem leaves) [108]. These results are consistent with the increase in the mRNA expression levels of the HMOX1 gene after EENL treatment. These results were validated by another study showing significant overexpression of HMOX1 RNA expression in HUVEC cells post treatment with 20.0 and 40.0 μg/mL of EENL for 24 h [108]. Yet another study evaluated compounds present in the leaves of the neem tree (*Azadirachta Indica*) as potential inhibitors for COVID-19 main protease (Mpro) (PDB code: 6LU7). The main protease (Mpro, also called 3CLpro) is an attractive drug target among coronaviruses because of its essential role in processing the polyproteins that are translated from the viral RNA. They used blind molecular docking using PyRx and Auto Vina software to compare the binding energies obtained from the docking of 6LU7 with *meliacinanhydride*, *nimocinol*, *isomeldenin*, *nimbolide*, *zafaral*, *nimbandiol*, *nimbin*, *nimbinene*, *desacetylnimbin* and *hydroxychloroquine* and *remdesivir* as positive controls. They found that *meliacinanhydride* (Ki = 33.36 pM) and the compounds from neem leaves had significantly high binding energy to 6LU7, which could point to a potential clinical drug option against COVID-19. Additionally, neem leaves also contain other immunity boosting compounds such as *quercetin*, *zinc*, *vitamin A*, *B1*, *B2*, *B6*, *C*, *E* [122,123].

**Figure 2:**
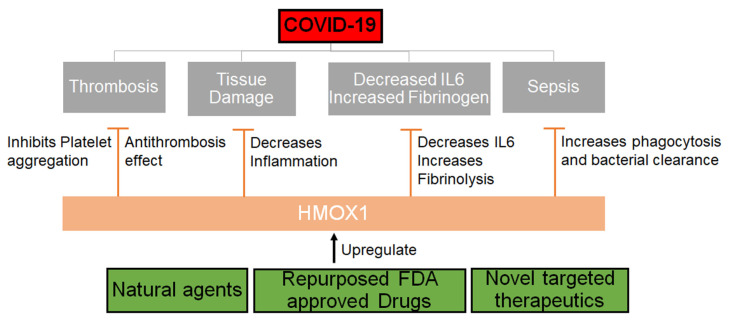
Mechanism through which HMOX1 can be regulated. HMOX1 that can be upregulated by either natural compounds or synthetic molecules may inhibit the effects of SARS-CoV-2 by decreasing inflammation, IL6 levels and increasing phagocytosis, fibrinolysis and thereby inhibiting thrombosis and sepsis.

To our understanding, this could be a great direction for further investigation of natural plants such as neem as potential therapeutic options for SARS-CoV-2 (Figure 2). Other agents that can upregulate HMOX1 should be explored and drugs that can be repurposed to upregulate or modulate the HMOX1 pathway should be urgently evaluated.

## 9. Conclusions

Infection with the SARS-CoV-2 virus leads to severe inflammation, causing damage to the lungs, blood vessels and organs. Paradoxically, the virus appears to stimulate the immune system in a manner that does not inhibit viral pathogenesis. Coronaviruses have likely evolved over millions of years in mammalian hosts such as bats, and it appears that the virus is able to evade the human immune system and prevent it from effectively clearing the infection in a significant subset of individuals. Here, we focus on the role of the human HMOX1 protein, which has an important anti-inflammatory role in attenuating serious conditions like thrombosis, sepsis, tissue damage and fibrinogenesis, all of which are associated with SARS-CoV-2 infections. The anti-inflammatory HMOX1 protein is bound by the SARS-CoV-2 open reading frame 3 a (ORF3a) protein; thus, we strongly suggest that this interaction is worthy of careful investigation. A very simple model is that viral ORF3a is able to inhibit the anti-inflammatory functions of HMOX1, and this leads to uncontrolled inflammation that is beyond the capacity of the human immune system to effectively counter. The story is likely much more complex, but studies to unravel the relevance of this interaction may lead to key insights into COVID-19 pathogenesis. Currently, we do not know the binding surfaces that interact, or the effect of inhibiting this binding on viral pathogenesis in mammals. While much effort has been focused on inhibiting the binding of the spike protein to the human ACE2 receptor, we propose that a similar effort should be made in developing ORF3a-HMOX1 inhibitors, if only as a tool to better understand the role of this interaction in COVID-19. Importantly, there are reports of recovered COVID-19 patients developing antibodies to the ORF3a protein [124,125], hinting that targeting it could play a role in inhibiting viral pathogenesis.

There are a number of approaches that could be used to investigate the role of HMOX1 and ORF3a in COVID-19 pathogenesis. For example, in animal models, does deletion of the ORF3a gene in the virus alter the inflammatory profile of lymphocytes in severe COVID-19 disease? If the binding surface between HMOX1 and ORF3a is determined, will gene variants that disrupt this interaction alter COVID-19 inflammatory severity? HMOX1 has a wide array of downstream targets that act to attenuate inflammatory signaling. Which signaling pathways are altered by heterologous expression of ORF3a in cell culture or lymphocyte models? For example, does overexpression of ORF3a alter the prevalence of M1 versus M2 macrophages? Although knockout of HMOX1 leads to a systemic pro-inflammatory phenotype in the knockout mouse model, would CRISPR knock-in of HMOX1 that retains wild-type function but lacks ORF3a binding affinity alter the progression of inflammation? There is currently a project at the University of California Davis mouse biology program to create the humanized mouse model for COVID-19 where mice express the human ACE2 gene which would allow this to be done in mice. Although the ultimate goal of developing drugs that upregulate HMOX1 or inhibit its binding to viral ORF3a is to treat severe disease, early candidates could be used as tools to determine the role of the HMOX1 pathway in COVID-19 inflammatory dysregulation.

Another line of questioning revolves around the role of neem derivatives. As the details of the COVID-19 inflammatory response is better defined, can the testing of neem products in animal models at different stages of disease lead to reduced inflammatory response to SARS-CoV-2 infection?

FDA-approved drugs such as BRD inhibitors, RNA translation inhibitors, sigma 1 and 2 receptor modulators and mTOR inhibitors are promising leads because they avoid prolonged FDA approval delays. Our article throws light on some drugs that can be repurposed and some that can be chemically synthesized specifically for desired targets. We also suggest natural compounds such as the neem plant that demonstrate an ability to upregulate HMOX1 in human cell lines. Therefore, we suggest future studies on natural and synthetic molecules that are focused on modulating the HMOX1 pathway as viable therapeutic options for SARS-CoV-2 infections and regulating innate immunity in patients.

## Figures and Tables

**Table 1:** Potential medications for the treatment of SARS-CoV-2.

Potential Drug	Mode of Action	Current Status	Reference
Remdesivir	Remdesivir (a variant of Adenosine) which hinders the coronavirus RNA polymerase—a key enzyme that coronavirus requires to replicate its genetic material and proliferate in a human body.	On 1 May 2020, the FDA issued an emergency use authorization (EUA) for remdesivir	[32,33]
Hydroxychloroquine and chloroquine	Hydroxychloroquine and chloroquine work via changing the chemical environment of human cell membranes, consequently not allowing the virus to enter and multiply inside the cells.	FDA has issued a caution that hydroxychloroquine (HCQ) or chloroquine should not be used outside of a hospital setting/clinical trials for COVID-19 due to serious heart-related side effects.	[34,35]
Azithromycin	Azithromycin is an antibiotic used to treat bacterial infections like bronchitis and pneumonia.	Azithromycin is FDA approved but should not be used in combination with hydroxychloroquine together due to serious side effect concerns.	[36,37,38,39,40]
Convalescent plasma and antibody-based therapies	Convalescent plasma is taken from people who have developed antibodies for COVID-19. This could potentially help fight the coronavirus infection in new patients. Synthetically produced antibodies can inhibit viral infection and pathogenesis.	The FDA issued an Emergency Investigational New Drug approval for the use of convalescent plasma to treat people with COVID-19 on 24 March 2020.	[41,42,43,44,45,46] (antibody refs)
Actemra (tocilizumab)	Actemra blocks interleukin-6 (IL-6), a cytokine involved in human immune response.	The efficacy and safety phase 2 trial of Tocilizumab for the treatment of COVID-19 is under way.	[19,47,48,49,50,51]
Kaletra (lopinavir/ritonavir)	KALETRA is a combination of lopinavir and ritonavir. Lopinavir is a potent inhibitor of the Human Immunodeficiency Virus (HIV) protease. Ritonavir obstructs the CYP3A-mediated metabolism of lopinavir, resulting in increased plasma levels of lopinavir.	A cluster randomized controlled trial (RCT) of oral Kaletra (lopinavir/ritonavir) as Post-Exposure Prophylaxis (PEP) is underway for COVID-19.	[52,53,54,55,56,57,58,59]
Tamiflu (oseltamivir)	Oseltamivir works by inhibiting the viral neuraminidase enzyme activity. The enzyme is found on the surface of the virus (H1N1).	Several ongoing clinical trials are looking at Tamiflu in combination with other medications for COVID-19.	[52,57]
Avigan (favipiravir)	Favipiravir/Avigan induces a rapid mutation rate of the virus RNA polymerase complex, which results in a large proportion of inactive viruses amongst the virus population.	Favipiravir/Avigan is an approved drug in Japan and China against flu. Clinical trial is underway in US to start in Boston.	[52,60,61,62]
Colcrys (colchicine)	Colchicine could work if the immune system becomes too activated and a cytokine storm occurs.		[63]

**Table 2:** Antiviral properties of neem.

Neem Plant Part	Virus Type	Reference
Aqueous extract preparation from the barks of neem (NBE)	Herpes Simplex Virus 1 (HSV-1)	[114]
Crude aqueous extract of neem leaves and pure neem compound (Azadirachtin)	Dengue virus type 2	[115]
Neem’s polysaccharides extracted from leaves	Poliovirus	[116]
Neem leaf extract	Coxsackie virus B-4	[117]
Water-extracted polysaccharides from neem leaves	Anti-bovine herpesvirus Type 1	[118]
Neem seed kernel extracts	Duck plague virus	[119]
Fractionated neem-leaf extract	Human Immunodeficiency Virus 1 (HIV-1)	[120]
